# Biodegradation of ochratoxin A by endophytic *Trichoderma koningii* strains

**DOI:** 10.1007/s11274-022-03491-2

**Published:** 2022-12-24

**Authors:** Ahmed A. Ismaiel, Hala H. Mohamed, Manal T. El-Sayed

**Affiliations:** Department of Botany and Microbiology, Faculty of Science, Zagazig University, Zagazig, 44519 Egypt

**Keywords:** Ochratoxin A (OTA) degradation, Dietary intake of OTA, *Aspergillus niger*, *Trichoderma koningii*, Carboxypeptidase A (CPA), FTIR, LC–MS/MS

## Abstract

Ochratoxin A (OTA) is a mycotoxin produced by *Aspergillus* spp. and *Penicillium* spp*.* that causes a threat to food safety and human health. Fungal biodegradation might be a promising strategy for reducing the OTA contamination in the future. In this study, the ability of *Trichoderma koningii* strains to degrade OTA produced by *Aspergillus niger* T2 (MW513392.1) isolated from tomato seeds was investigated. Among *T. koningii* strains tested, three strains; AUMC11519, AUMC11520 and AUMC11521 completely eliminated OTA from the culture medium, while AUMC11522 strain eliminated only 41.82% of OTA. OTα-amide, 3-phenylpropionic acid, OTα and phenylalanine were assayed as degradation products by FTIR analysis and LC–MS/MS spectra. Carboxypeptidase A (CPA) was found responsible for OTA degradation when a metal ion chelator, EDTA, was added to cell free supernatants of the three effective strains. OTA detoxification by *T. koningii* could present new prospective strategies for a possible application in food commodities intoxicated with ochratoxin.

**Supplementary Information:**

The online version contains supplementary material available at 10.1007/s11274-022-03491-2.

## Introduction

Ochratoxin A (L-phenylalanine-*N*-[(5-chloro-3,4-dihydro-8- hydroxy-3-methyl-1-oxo-1H-2-benzopyran-7-yl)carbonyl]-(*R*)-isocoumarin) (OTA, Supplementary Fig. S1), is a mycotoxin, which was first discovered and chemically characterized by van der Merwe et al. (1965a, b) in a South African culture of *Aspergillus ochraceus* inoculated in a corn meal. Subsequently, it was found to be produced by different species of *Aspergillus* and *Penicillium*, particularly *A. ochraceus, A. niger* and *P. verrucosum*, respectively (Ali et al. 2013; García-Cela et al. 2014; Ismaiel and Papenbrock 2015).

OTA contaminates a wide range of food and feed commodities in many countries, such as cereals, cereal products, grapes, coffee, dried vine fruits, grape juice, nuts, spices and animal-derived food stuff (Duarte et al. 2012). OTA causes economic losses when synthesized in pre- and post-harvest plants as well as the losses occurred when synthesized in stored products, it also causes chlorosis of leaves, increased oxidative stress and even causes cell death of plants (Hao et al. 2015). OTA phytotoxicity is associated with damage of DNA, inhibition of protein synthesis, generation of reactive oxygen species, dysfunction of mitochondria, and the calcium homeostasis disruption (Wang et al. 2014). It also enhances free radical production, which may affect the redox-regulated antioxidant activity of antioxidant enzymes including catalase, glutamate cysteine ligase, superoxide dismutase, glutathione peroxidase, glutathione and glutathione-S-transferases (Preuss et al. 2014).

Previous studies showed that OTA has nephrotoxic, hepatotoxic, neurotoxic, teratogenic, mutagenic and immunotoxic characters and it is capable of causing kidney and liver tumors in mice and rats, its toxicity is dependent upon sex, the cellular type and the species of the tested animals (Abrunhosa et al. 2010). It was reported that OTA causes the formation of DNA-adducts after chronic exposure of OTA to rat and sub-acute exposure to pig (Faucet et al. 2004). OTA is also classified as possibly carcinogenic (group 2B) to humans as there is evidence of its carcinogenicity on experimental animals not on humans (IARC 1993). Due to all these health hazardous effects, OTA was subjected to legal regulations on both national and international levels by the World Health Organization, (WHO), which proposed the maximum level for OTA in cereals of 5 µg kg^−1^ (WHO 1991). Moreover, the European Union member states set new limits for dietary intake of OTA ranged between 15 and 60 ng kg^−1^ bw per week for adult consumers (European Food Safety Authority, EFSA, 2006).

The particularity of OTA is due to its high stability, as it is partially degraded during cooking conditions (Müller 1982)**,** and it resists the food processing temperature. So that, it can be detected in processed food products such as wine, beer and bread (Duarte et al. 2010). It can resist 3 h of steam sterilization at 121 °C (Trivedi et al. 1992), and even at 250 °C, the destruction of the toxin is not complete (Boudra et al. 1995). It is also highly resistant to conventional treatment processes such as thermal sterilization and fermentation (Bullerman and Bianchini, 2007; Kabak 2009).

A considerable attention has been paid for the biodegradation of OTA, either in food products or in aqueous solutions. Cell cultures of plants (wheat, maize, tomato, soybean, sweet potato tubers) completely transformed OTA into a number of other products (Karlovsky 1999). *Streptococcus salivarius* subsp. *thermophilus*, *Bifidobacterium bifidum*, and yogurt bacteria have eliminated OTA levels in milk samples containing 0.05 and 0.1 mg OTA L^−1^; *Lactobacillus delbrueckii* subsp. *bulgaricus* reduced OTA level in milk samples containing 0.5 mg OTA L^−1^ (Škrinjar et al. 1996). In literature, some filamentous fungi showed potentiality of OTA biodegradation. *A. fumigatus*, *A. japonicus* and *A. niger* completely degraded 2 mg OTA L^−1^ after 10 days of incubation at 30 °C, it was degraded into OTα and further degradation into an unknown compound was observed (Varga et al. 2000). OTA was partially or completely degraded by *A. niger* and other filamentous fungi, and OTα was detected, especially in the assays carried out by *A. niger* and other black fungi (Abrunhosa et al. 2002). *A. ochraceus* (OTA non-producer), and some strains of *A. wentii* completely degraded OTA producing unidentified degradation metabolites (Varga et al. 2000). This study presents new insights on the biodegradation of OTA using endophytic strains of *T. koningii* to less- and non-toxic compounds. This is promising in food and agricultural application for the minimizing the toxicity of OTA.

## Materials and methods

### Chemicals and OTA standard

All chemicals and solvents used in this study were of high degree of purity. The standard OTA was obtained from Sigma-Aldrich, Taufkirchen, Germany. HPLC-grade solvents such as *n-*hexane, methylene chloride, chloroform and methanol were used for extraction procedures and thin layer chromatographic (TLC) analysis. Acetonitrile, methanol and citric acid monohydrate used for HPLC analysis, were purchased from Merck, Darmstadt, Germany.

### Fungal strains used in the current study

#### Ochratoxigenic fungus

*Aspergillus niger* T2, was isolated from a tomato sample (*Solanum lycopersicum* L.), obtained from a retail market in Sharkia governorate, Egypt. It was isolated by dilute plate method on potato-dextrose agar (PDA) (Nazir et al. 2014). The fungal isolate was molecularly identified based on the sequence of PCR-amplified ITS1-5.8S and ITS4 rRNA-gene analysis performed at The Animal Health Research Institute, Dokki, Giza, Egypt. Sequence was further analyzed using Basic Local Alignment Search Tool (BLAST) program (Altschul et al. 1997) from the National Center of Biotechnology Information (NCBI) website. The sequence size of the fungal strain was successfully deposited in the Genebank with accession number MW513392.1.

#### Trichoderma koningii strains used in the biodegradation assay

Four endophytic *T. koningii* strains were used for OTA detoxification activities. These are *T. koningii* CTX1185, *T. koningii* CTX1172, *T. koningii* TD5391 and *T. koningii* TR2715. The first and second strains were previously isolated from *Cupressus macrocarpa* twig, while the third and fourth strains were isolated from the bark of *Terminalia distichum* and *T. arjuna*, respectively. They were morphologically identified and deposited in the Assiut University Mycological Center (AUMC, http://www.aun. edu.eg/aumc/aumc.htm) with accession numbers: AUMC11519, AUMC11520, AUMC11521, and AUMC11522, respectively (Ismaiel and Ali 2017).

### Inoculum preparation and growth conditions

The ochratoxigenic fungal strain, *A. niger* MW513392.1 was cultivated in 250 mL Erlenmeyer flasks containing 50 mL PD broth with pH 5.6 at 30 °C. For inoculum preparation, fungal conidia from 7-day old cultures of the strain were harvested in sterile distilled water containing 0.01% Tween 80, and gently scrapped off with a sterile glass rod. The spore suspensions were then adjusted to final concentrations of 10^6^ conidia mL^−1^ using a hemocytometer. One mL of the freshly prepared inoculum of the fungal strain was added to the Erlenmeyer flasks containing medium. Culture flasks were then dark-incubated at static conditions for 10 days at 30 °C.

### Extraction and purification of OTA

Extraction of OTA was performed in two steps. Firstly, the culture filtrate of *Aspergillus niger* T2 (MW513392.1) was defatted with *n*-hexane, after which OTA was extracted with an equal volume of methylene chloride, then the mixture was shaken for 30 min and allowed to stand for 30 min in a separating funnel. The methylene chloride layer was filtered over anhydrous sodium sulfate and then evaporated under a vacuum to dryness (Daradimos et al. 2000; Téren et al. 1996; Valenta et al. 1993). OTA samples were then purified using column chromatography. The dried extract was dissolved in 0.01 M HCl (5 mL), filtered through glass microfiber filter (55-mm diameter GF/A, Whatman, Maidstone, UK). The solution was subjected to solid-phase extraction column (SPE) (Al-Hadithi et al. 2015). The packing material used in the SPE column was silica gel, C18 bonded to silica gel (Zheng et al. 2006). A rubber syringe plunger was used to push the sample extract through the SPE column which retains the impurities and purified extract was collected in a test tube (Malone et al. 1998).

### Determination of OTA

#### TLC

In order to detect OTA, the TLC plates were prepared according to Stahl (1969). Ten grams of silica powder GF-254, purchased from Sigma-Aldrich, Taufkirchen, Germany, were mixed with 30 mL warm distilled water with continuous stirring till the formation of a slurry, then poured on a glass plate (20 × 20 cm) and allowed to solidify at room temperature. The plates were kept in an electric oven at 110 °C for 1 h in a vertical position, then used immediately. OTA was then detected qualitatively according to Téren et al. (1996). The dried crude extract was re-dissolved in absolute methanol (250 μL), spotted on TLC plates along with OTA standard solutions using glass capillary tubes approximately 2 cm away from the bottom and 2 cm away from the edges of the plate and in-between the spots. After that, the TLC plates were placed into a solvent tank containing chloroform: methanol (93:7, v/v) as a developing system. The solvent was removed when rises up and reaches approximately 2 cm from the end of the plates. The developed TLC plates were allowed to dry at room temperature. After which, OTA spots of samples and standard were visualized as greenish-blue fluorescence under UV (366 nm). The rate of flow (Rf) of OTA is calculated by using the following formula (Snyder 2008):

$${\text{Rf}} = {\text{ Distance traveled by compound }}\left( {{\text{Ds}}} \right)/{\text{ Distance traveled by the solvent front }}\left( {{\text{Df}}} \right)$$

The spots of both samples and standard OTA scrapped off, eluted in 3 mL methanol, and centrifuged at 2516 ×g for 10 min (Nesheim 1976). The OTA supernatants were estimated using a UV spectrophotometer (6800UV/ Vis. Spectrophotometer, Jenway) at 365 nm against 3 mL of methanol as control and concentrations were obtained from a standard curve (Nesheim 1976).

#### HPLC

HPLC analysis was performed at Animal Health Research Institute, Dokki, Giza, Egypt, using Agilent Series 1200 quaternary gradient pump, Series 1200 auto sampler, Series 1200 FLD detector, and HPLC 2D Chemstation software (Hewlett-Packard, Les Ulis, Germany). The chromatographic separation was performed using a reversed-phase column (Extend-C18, Zorbax column, 4.6 mm i.d., 250 mm, 5 μm, Agilent Co.), in which the mobile phase should be sufficiently transparent at the wavelength of detection. The mobile phase used was water: acetonitrile: methanol (60: 20: 20, v:v:v) (Durguti et al. 2014). The fluorescent detector was set to a wavelength of 330 nm for excitation and 460 nm for emission. The column temperature adjusted at 30 °C at a flow rate of 1.0 mL min^−1^ to achieve the optimum resolution of the OTA. The injection volume was maintained at 20 μL for both sample and standard. Calibration curve was prepared using different concentrations of OTA standard. The linearity of detector response for the standard was determined by means of linear regression.

### OTA concentration used in degradation tests

The chromatographically separated OTA from *A. niger* MW513392.1 was pooled and dissolved in chloroform (AnalaR) at concentration of 10 mg mL^−1^ and stored in the dark at – 20 °C. In order to test the biodegradation process of OTA, the chloroform was evaporated and the crystalline OTA was dissolved in 5 mL of DMSO and the dilution was made to achieve an initial OTA concentration of 5 µg mL^−1^ (Wei et al. 1985).

### Degradation assay of OTA

*T. koningii* strains (AUMC11519, AUMC11520, AUMC11521 and AUMC11522) were first grown in PDA for 7 days in the dark at 30 °C for inoculum generation. The fungal strains were then grown in test tubes containing 3 mL of PDB amended with 5 µg mL^−1^ of OTA. Test tubes were inoculated with a dense conidial suspension of the *T. koningii* strains, the spore suspension was adjusted to a concentration of 10^6^ spores mL^−1^ and incubated at 30 °C for 10 days in the dark. After which, the fungal cultures were filtered by Whatman No. 1 filter papers. A negative control, a culture medium-containing OTA, was used to calculate OTA removal percentage. All assays were performed in triplicate (Varga et al. 2000). OTA residues were extracted and quantified spectrophotometrically as mentioned earlier.

$${\text{Removal }}\% \, = \, \left( {{\text{C}}_{0} {-}{\text{ C}}/{\text{ C}}_{0} } \right) \times {1}00$$

where C_0_ and C represented the initial and residual concentrations of OTA, respectively (Bejaoui et al. 2006).

### Effect of EDTA on OTA degrading activity

*T. koningii* strains were grown separately in PD broth for 7 days at 30 °C. The cells from the 50-mL *T. koningii* cultures were harvested by centrifugation at 6440 ×g at 4 °C for 20 min. The supernatant was collected and processed by surface sterilization to produce a cell-free supernatant by filtering 0.22 µm filters. Thereafter, the cell-free supernatants were amended with 5 and 10 mmol^−1^ of EDTA. The EDTA-free broth served as a control (Zhang et al. 2017). In all of these assays, the initial concentration of OTA was 5 µg mL^−1^.

### Fourier-transform infrared (FTIR) analysis

The filtrate of both samples and controls resulted from degradation assays processed above were analyzed by FTIR. In which, the filtrates were extracted with chloroform and the dry films were stored at − 20 °C till the analysis by FTIR which was performed at Microanalytical Center, Faculty of Science, Cairo University, Giza, Egypt. Infrared spectra of treated and non-treated OTA resulted from detoxification experiments were determined over the region 400–4000 cm^−1^ with Pelkin-Elmer FTIR 1650 spectrophotometer.

### Liquid chromatography-mass spectroscopy/ mass spectroscopy (LC–MS/MS) analysis

To distinguish the products of OTA biodegradation by the most efficient *T. koningii* AUMC11521, LC–MS/MS was performed with compound-specific modification to compare the fluorescent and mass transitional chromatograms. The fluorescent detector was set to a wavelength of 333 nm for excitation and 460 nm for emission. LC–MS/MS was performed in the Regional Center for Food and Feed (RCFF), Agricultural Research Center, Giza, Egypt. The analysis was performed using Agilent 1200 series liquid chromatography system equipped with Applied Biosystems (API 4000 Qtrape) tandem mass spectrometers with electrospray ionization (ESI) interface. Agilent 1200 series liquid chromatography system was equipped with Applied Biosystems (API 4000 Qtrape) tandem mass spectrometers with electrospray ionisation (ESI) interface. Separation was performed on a C18 column ZORBAX Eclipse XDBC18 4.6 mm × 150 mm, 5 μm particle sizes. The injection volume was 25 μL. A mobile phase was at 0.3 mL/min flow rate, in which one reservoir contained 10 mM ammonium format solution in methanol: water (1:9, v/v). The ESI source was used in the positive mode, and nitrogen was used as nebulizer gas, curtain gas, heater gas and collision gas according to manufacturer’s settings; source temperature was 300 °C, ion spray potential 5500 V. Declustering potential and collision energy were optimized by using the Harvard apparatus syringe pump. The Multiple Reaction Monitoring Mode (MRM) was used in which one MRM was used for quantification and other was used for confirmation (qualifier peaks).

### Statistical analysis

Data obtained were subjected to the statistical analysis of variance according to Snedecor and Cochran (1980), and means separation were done according to Duncan (1955).

## Results

### Production and purification of OTA from *A. niger* T2 (MW513392.1)

**Fig. 1:**
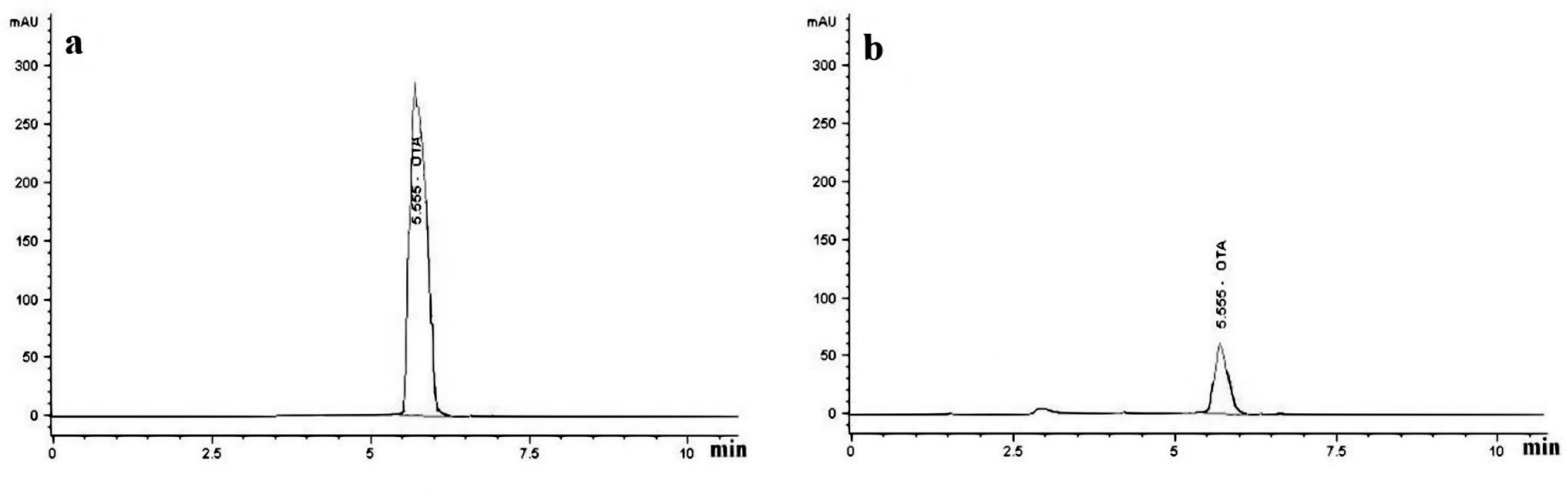
HPLC chromatograms of OTA with a single peak at a retention time of 5.555 min. **a** Standard. **b** OTA sample separated from *A. niger* MW513392.1 after cultivation in PDB for 10 days at 30 ºC

The ochratoxigenic strain, *A. niger* T2, was isolated from a tomato seeds sample collected from a retail shop during a survey of fungal contamination. Based on the colony appearance, morphological criteria, and conidial arrangement, the isolate was identified. It was further molecularly identified and its sequence (480 pb) has been deposited in GenBank with accession number MW513392.1. The strain was tested for the production of OTA in PDB after incubation for 10 days. The chloroform extract of the fungal filtrate showed the presence of OTA spot on TLC at Rf value = 0.89. The fungal strain was found to produce OTA in amount up to 86 µg L^−1^ using PDB after 10 days of incubation. After purification with column chromatography, the purified OTA using HPLC analysis gave a single peak at a retention time of 5.555 min that was identical with the standard peak (Fig. 1a, b). The recovered OTA was used later in the biodegradation studies by *T. koningi* strains*.*

### OTA degrading activity of *T. koningii* strains

**Fig. 2:**
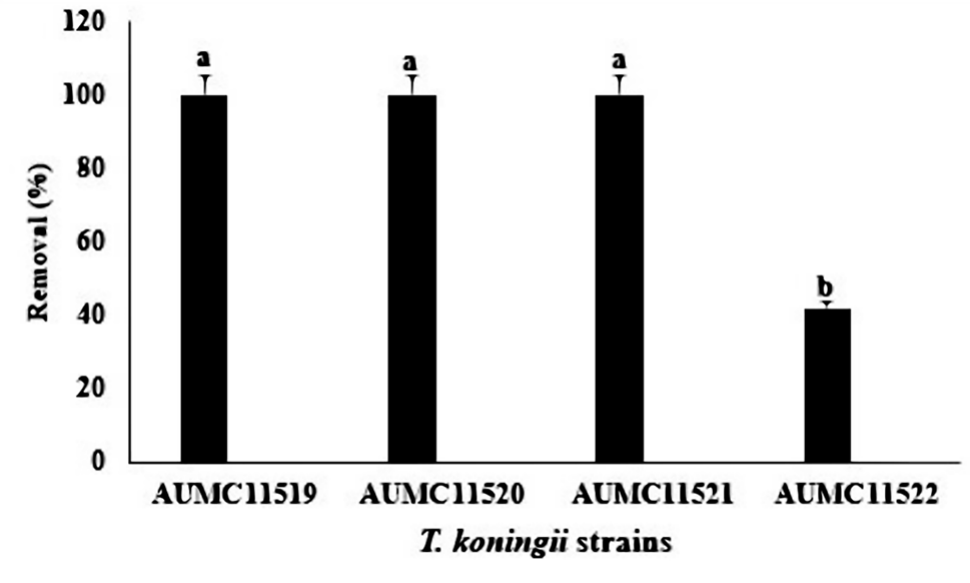
Biodegradation of OTA by *T. koningii* strains after incubation for 10 days at 30 °C under static conditions. Data was expressed in percentage of OTA removal. Values are represented as means ± SD of three replicate analyses from two independent experiments. Different letters on the bars for OTA removal (%) indicate significant differences

Four endophytic strains of *T. koningii* (AUMC11519, AUMC11520, AUMC11521 and AUMC11522) were inoculated individually in PDB spiked with OTA (5 μg mL^−1^) and incubated for 10 days at 30 °C in order to demonstrate the biodegradation activity. Data presented in Fig. 2 showed that three strains, AUMC11519, AUMC11520 and AUMC11521 completely eliminated OTA from the culture medium in a percent of 100%, while AUMC11522 strain eliminated only 41.82% of OTA recording significant differences (*P* ≤ 0.05) with the other test strains.

### Effect of EDTA on OTA degrading activity

**Fig. 3:**
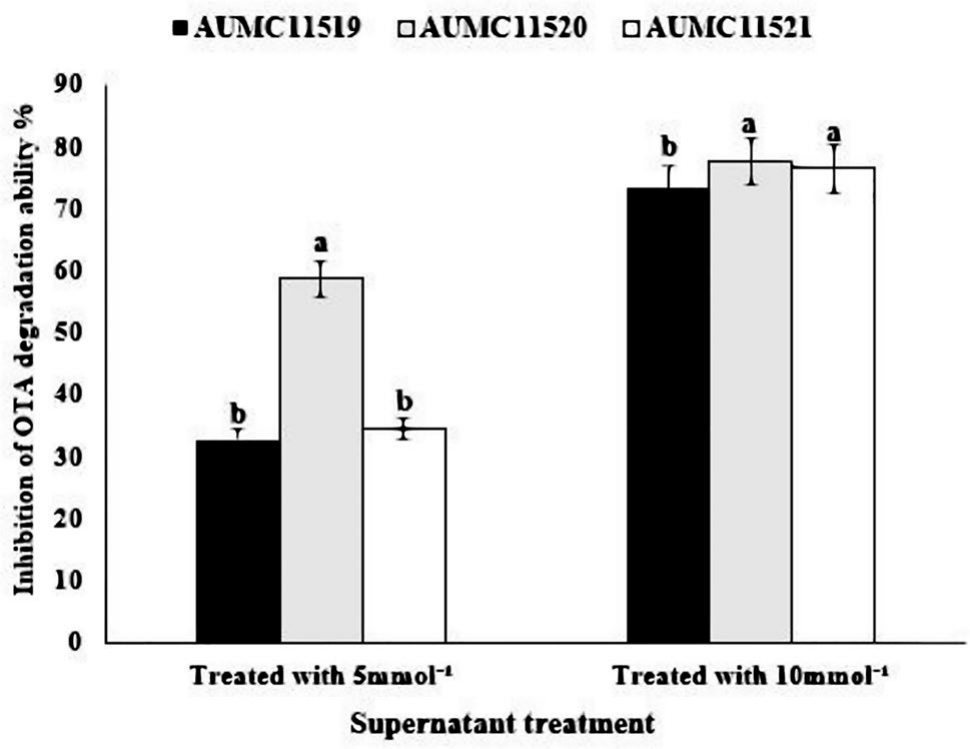
Effect of cell-free supernatants of *T. koningii *strains (AUMC11519, AUMC11520, and AUMC11521) treated with 5 and 10 mmol^−1^ EDTA on OTA removal%. Samples were incubated at 30 °C for 7 days with 5 μg OTA mL^−1^. Values were represented as means ± SD of three replicate analyses from two independent experiments. Different letters on the bars for either treatment with 5 mmol^−1^ EDTA or 10 mmol^−1^ indicate significant differences

*T. koningii* strains were grown separately in PD broth for 7 days at 30 °C. After centrifugation, the cell-free supernatants were supplemented separately with 5 and 10 mmol^−1^ EDTA. The EDTA-free supernatants of the three test strains showed a total 100% effective removal of OTA. EDTA-treated supernatants showed a dramatic significant reduction in the percent of OTA removal ability (*P* ≤ 0.05) (Fig. 3). The 5 mmol^−1^ EDTA-amended supernatants of the three strains inhibited OTA degradation ability by 58.59% (AUMC11520), 34.3% (AUMC 11,521), and 32.68% (AUMC11519). Upon treatment with 10 mmol^−1^ EDTA, the inhibition of degradation efficiency (%) was significantly reduced by 77.57% (AUMC11520), 76.34% (AUMC11521) and 73.15% (AUMC11519).

### Characterization of OTA biodegradation by the most potent *T. koningii* strains.

#### FTIR

**Fig. 4:**
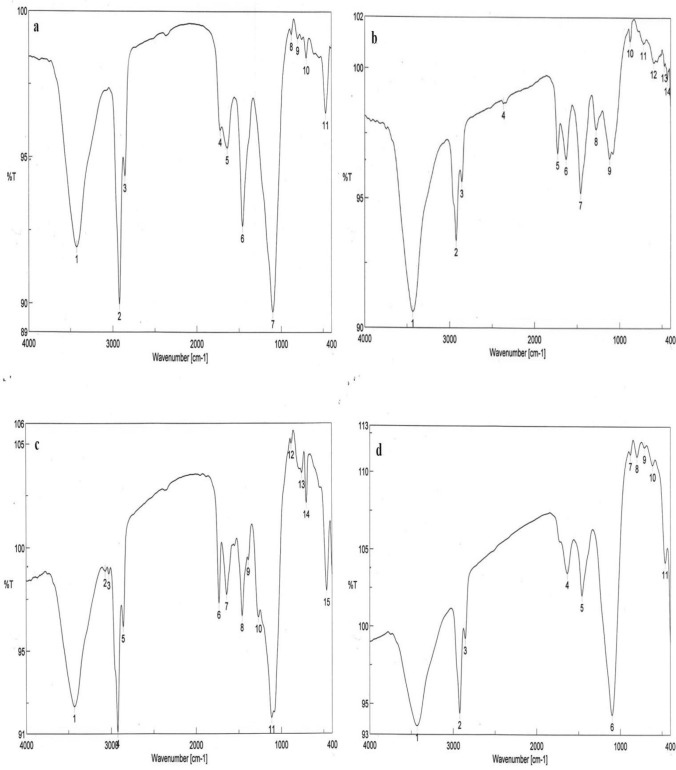
FTIR spectra of filtrate samples of *T. koningii* strains after incubation with OTA (5 µg mL^−1^) for 7 days at 30 ºC. **a** Control OTA (5 µg mL^−1^). **b** AUMC11519 strain. **c** AUMC11520 strain. **d** AUMC11521 strain

To estimate the structural changes of the biodegraded OTA, the FTIR spectra of OTA before and after incubation for 7 days at 30 °C with *T. koningii* AUMC11519, AUMC11520, and AUMC11521 were analyzed (Fig. 4a–d, respectively). According to FTIR spectrum of OTA treated with AUMC11521 strain (Fig. 4d), shifts at 3426.9 cm^−1^, 1640.2 cm^−1^ (∆ 6 cm^−1^), and 699.1 cm^−1^ (∆ 10 cm^−1^) coupled with the disappearance of peak at 1722.1 cm^−1^ and new peak at 612.3 cm^−1^ confirmed the cleavage of C=O of carboxylic acid and C=O of amide bond NH–CO and=C–H with C–Cl. FTIR spectra of OTA treated with AUMC11519 strain (Fig. 4b) and AUMC11520 strain (Fig. 4c) showed a shift at 1722.1 cm^−1^ (∆ 6 cm^−1^). New peaks at 1278.6 cm^−1^ (AUMC11519 strain) and 1386.6 cm^−1^ (AUMC11520 strain) are assigned to stretching mode of C–O (ether and carboxylic acid), C–F, NO_2_ (aliphatic nitro) and binding mode of C–O–H, respectively. A remarkable shift at 799.4 cm^−1^ (∆80 cm^−1^) in the case of AUMC11519 strain attributed to the bending mode of=C–H (aromatic ring), and N–H, and C–Cl stretching mode in OTA structure. From the results of FTIR spectra of *T. koningii* strains incubated with OTA, the AUMC11521 strain had incurred more changes in OTA degradation, compared with the other strains. Therefore, further characterization of products resulted from OTA degradation by this strain was proceeded by LC–MS/MS.

#### Confirmation of OTA biodegradation products by LC–MS/MS

**Fig. 5:**
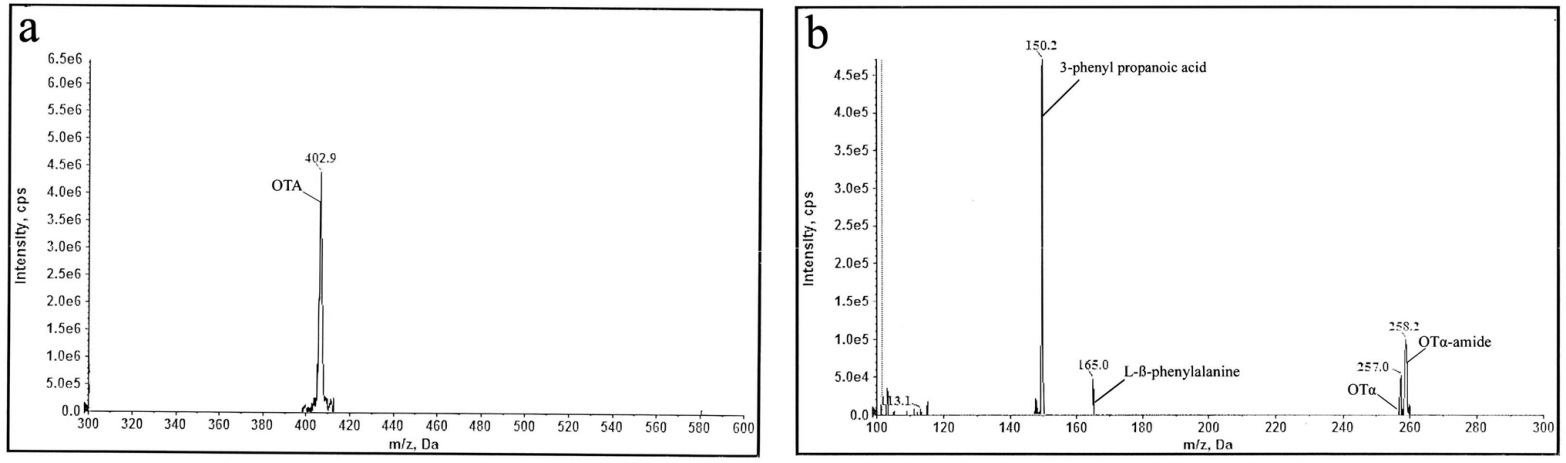
LC–MS/MS spectra of OTA-biodegraded products. **a** Control OTA **b** OTA after incubation with *T. koningii* AUMC11521 grown in PDB for 7 days at 30 °C

**Fig. 6:**
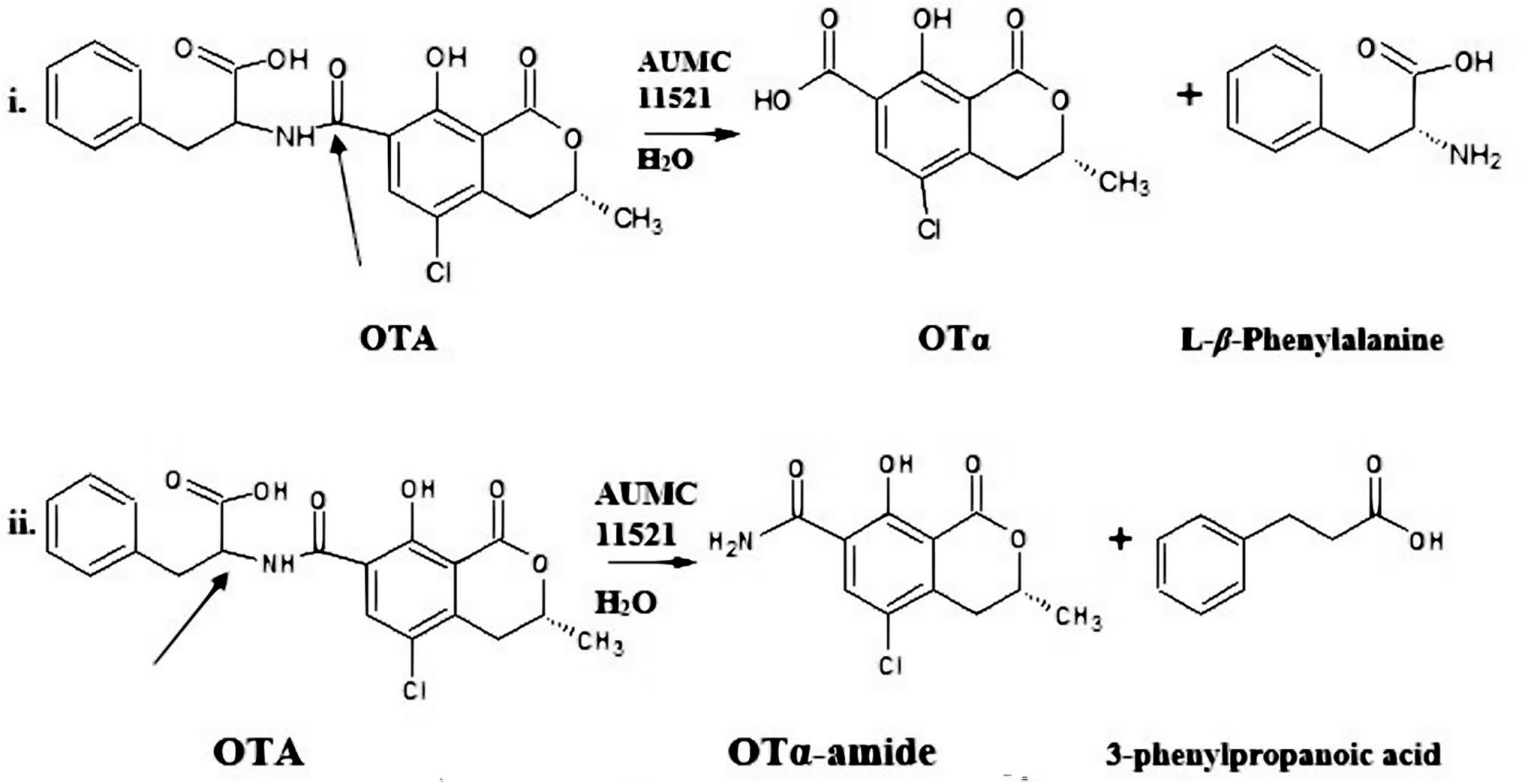
Biodegradation of OTA by *T. koningii* AUMC11521 grown in PDB for 7 days at 30ºC. i Ochratoxinases with amido-hydrolase activity are produced to break the amide bond in OTA, releasing two non-toxic products: OTα and L-β-phenylalanine; ii Hydrolases also produce less toxic metabolites: OTα-amide and 3-phenylpropanoic acid. Arrows refer to the positions of the cleavage of the bond responsible for the breakdown of OTA molecule

In order to identify the biodegradation products resulted from incubation of OTA with the most efficient OTA degrading strain (*T. koningii* AUMC11521) compared with control OTA (before incubation), LC–MS/MS analyses were employed (Fig. 5). Based on the LC–MS/MS spectra, the detected m/z 402.9 (Fig. 5a), m/z 258.2, m/z 257.0, m/z 165.0, and m/z 150.2 (Fig. 5b) were assigned to the separated OTA, OTα-amide, OTα, L-*β*-phenylalanine, and 3-phenylpropanoic acid, respectively. Their chemical formula were C_20_H_18_ClNO_6_ (OTA), C_11_H_12_ClNO_4_ (OTα-amide), C_11_H_9_ClO_5_ (OTα), phenylalanine (C_9_H_11_NO_2_), and C_9_H_10_O_2_ (3-phenyl propanoic acid) (Table 1). These changes confirmed the two hypothesized biodegradation pathways of OTA (Fig. 6). This is in accordance with the finding of FTIR studies which confirmed the cleavage of C=O of carboxylic acid and C=O of NH–CO and=C–H with C–Cl. The degradation products with their molecular weight (MW), chemical formula, and structural formula were presented in Table 1.

**Table 1:** The molecular weight (MW), chemical formula, and structural formula of OTA, and the biodegradation products analyzed by LC–MS/MS after incubation of OTA with *T. koningii* AUMC11521 for 7 days at 30 ºC

No	MW	Compound name	Chemical formula	Structural formula
1	402.9	OTA	C_20_H_18_ClNO_6_	
2	258.2	OTα-amide	C_11_H_12_ClNO_4_	
3	257.0	OTα	C_11_H_9_ClO_5_	
4	165.0	L-β-phenylalanine	C_9_H_11_NO_2_	
5	150.2	3-phenylpropanoic acid	C_9_H_10_O_2_

## Discussion

The toxic metabolites of storage molds are known as mycotoxins and the health hazards caused by them are known as mycotoxicosis. The entire problem of mycotoxins production and health hazards in humans were well documented (Klich et al. 2003; Trucksess et al. 2003). Due to OTA toxicity on human and animal health, this toxin is not allowed to be present above maximum permitted levels in agricultural products intended to be used as foods or animal feed. Substantial efforts have been exerted to study the critical points of OTA presence in food and feed commodities, and its detoxification methods have also been investigated (Abrunhosa et al. 2010). Although it is almost impossible to entirely prevent the formation of OTA in all cases, OTA accumulation can be minimized. A remarkable attention has been paid regarding demonstrating the effective methods for detoxification of mycotoxins contaminated commodities. Decontamination or detoxification is a pressing issue; this is useful in order to recondition mycotoxin contaminated agricultural products for use as animal feeds. Although certain treatments have been found to reduce levels of specific mycotoxins, however no single method has been developed that is equally effective against the wide variety of mycotoxins which may co-occur in different commodities (Abrunhosa et al. 2010). Regarding OTA, the most promising approaches included the use of microbes or their enzymes for decontamination purposes (Abrunhosa et al. 2010).

**Fig. 7:**
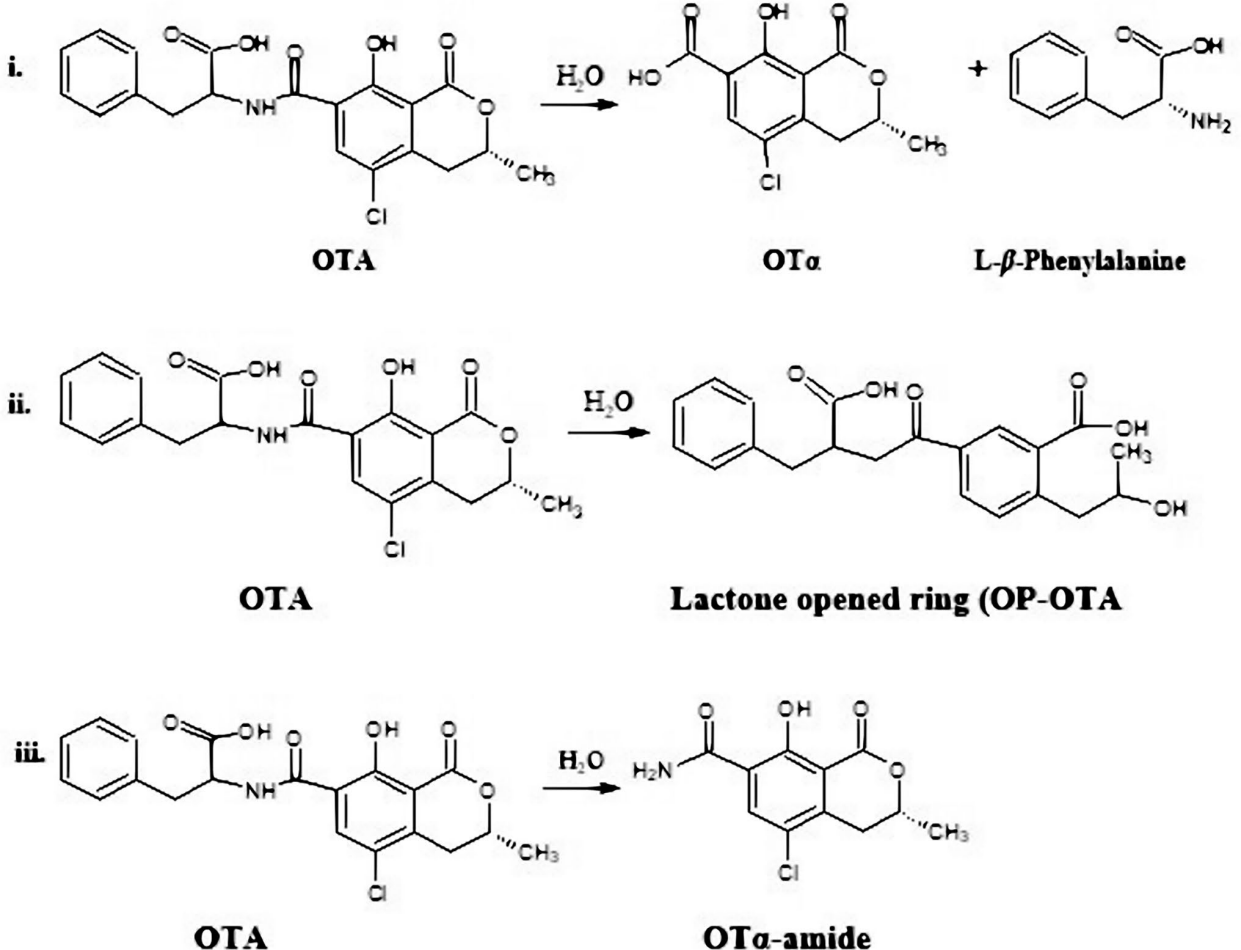
OTA degradation. i and ii Enzymatic biodegradation of OTA (Abrunhosa et al. 2010); iii Thermal degradation (Bittner et al. 2015)

Biodegradation or biodetoxification of OTA by fungi include the application of microbes or their enzymes for decontamination purposes (Abrunhosa et al. 2010). There are two different biodegradation pathways of OTA by enzymes. First biodegradation pathway occurred through the hydrolysis of the amide bond resulting in the production of L-*β*-phenylalanine molecule and OTα (Fig. 7i), which are non-toxic, so that this process is considered detoxification of OTA (Abrunhosa et al. 2010; Karlovsky 1999). The second pathway, biodegradation occurred through the hydrolysis of the lactone ring, resulting in the production of open ring OTA (Fig. 7ii), which have the same toxicity as OTA, so that this process is not considered as detoxification but it is just break down of OTA molecules (Abrunhosa et al. 2010; Karlovsky 1999). There is a third hypothetical pathway that was mostly observed during thermal degradation of OTA, in which the degradation occurred through the hydrolysis of the carbonyl of carboxylic acid resulting in the formation of the non-toxic OTα-amide (Fig. 7iii), this process is also considered detoxification (Bittner et al. 2015).

The current study showed a degradation percent ranged from 41.82 ± 1.57% to 100% of OTA with *T. koningii* strains. Similarly, *Trichoderma* spp. were able to degrade more than 80% of aflatoxin B1 (Shantha 1999). These differences in the biodegradation values occurred due to the strain-specificity (Petruzzi et al. 2015). *Trichoderma* spp. were previously used to inhibit OTA production by different ochratoxigenic fungi and suppress the growth of these fungi by production of certain enzymes as a result of antagonism (Vankudoth et al. 2016). In literature, no reports regarding using *T. koningii* strains in biodegradation of OTA are found, but their OTA-degrading activities may be attributed to the ability of *Trichoderma* spp.to produce extracellular enzymes such as hydrolases (Patil et al. 2016), proteolytic enzymes (proteases) (Aziz et al. 1993; Schuster and Schmoll 2010), including carboxypeptidase A (CPA) (Kupski et al. 2018). Therefore, *Trichoderma* spp. have a major impact on the biological control potential (Viterbo et al. 2002). CPA belongs to extracellular proteases and produced by *Trichoderma* spp. was found to be responsible for OTA degradation (Abrunhosa et al. 2002, 2006; Abrunhosa and Venâncio 2007; Schuster and Schmoll, 2010; Kupski et al. 2018).

In order to prove that extracellular CPA is responsible for OTA degradation, the cell-free supernatants of *T. koningii* strains (AUMC11519, AUMC11520, and AUMC11521) were amended with 5 and 10 mmol^−1^ of EDTA. Results showed a dramatic reduction in the OTA elimination ability of *T. koningii* strains. Based on previous studies concerning enzyme inhibition data (Abrunhosa and Venâncio 2007; Zhang et al. 2017), the OTA degrading enzyme from *T. koningii* strains was strongly inhibited by EDTA, demonstrating that the enzyme produced by *T. koningii* strains is a metalloenzyme. These results strongly suggest that *T. koningii* strains produce an enzyme that is responsible for biodegradation of OTA through the hydrolysis of carbonyl C-14 and the nitrogen atom of amine group, which resulted in the production of OTα-amide. In agreement with our results, studies carried out by Abrunhosa et al. (2002) on other filamentous fungi including *A. niger* showed completely or partially degradation of OTA after growth in 1 mg L^−1^ OTA for 6 days at 25 °C, OTα was also detected as a degradation product in their study. The authors concluded that a carboxypeptidase may be involved in the degradation of OTA by their strains. In a previous study (Zhang et al. 2017), *Alcaligenes faecalis* isolated from soil samples was found to degrade OTA efficiently and OTα was confirmed as a degradation product in the intracellular extract of *A. faecalis* using UPLC-MS/MS. It was suggested that the biodegradation of OTA occurs through the hydrolysis of the OTA amide bond by a putative peptidase (Zhang et al. 2017). In the current study, the OTA degradation by *T. koningii* strains released OTα-amide, 3-phenylpropionic acid, OTα and phenylalanine, indicating breakage of the amide bond and suggesting that the possible mechanism involved in OTA degradation by *T. koningii* strains is enzymatic.

Enzymatic degradation of OTA has specific advantages (Zhao et al. 2020). First, the degradation products have low toxicity. Second, OTα is a non-toxic compound with a tenfold shorter half-life time in humans (Bui-Klimke and Wu 2015; Zhao et al. 2020) and is 1000 times less toxic than OTA (Rodriguez et al. 2011). Compared with adsorption, degradation has obvious advantages. Hence, the conversion processes of OTA into OTα contribute substantially to reducing the toxic effects of OTA and are considered promising routes for OTA detoxification. Additionally, the enzymatic degradation is a non-reversible process that does not cause secondary pollution. Cell wall adsorption by microorganisms such as yeast and lactic acid bacteria does not completely eliminate OTA, and there are still hidden dangers of desorption (Zhao et al. 2020).

The FTIR spectra were carried out to identify the changes occurred in OTA structure in the range of 4000–400 cm^−1^. The position or the intensities of peaks of OTA are expected to be changed upon this interaction. On the basis of FTIR spectra of OTA (before and after biodegradation) *T. koningii* AUMC11521 was found to be the most effective strain in OTA degradation. FTIR spectrum of OTA treated with this strain showed shifts at 3426.9 cm^−1^, 1640.2 cm^−1^ (∆ 6 cm^−1^), and 699.1 cm^−1^ (∆ 10 cm^−1^) coupled with the disappearance of peak at 1722.1 cm^−1^ and new peak detected at 612.3 cm^−1^ confirmed the cleavage of C=O of carboxylic acid and C=O of amide bond NH–CO and=C–H with C–Cl. These changes in OTA molecules are assigned to the -OH and -NH groups and hydrogen bonding in fingerprint region between 2500 cm^−1^ and 3800 cm^−1^ which corresponds to protein component (Bhat, 2013). Therefore, OTA biodegradation products by the ultraefficient strain AUMC11521 were subjected to further characterization by LC–MS/MS.

LC–MS/MS analyses of fungal OTA before and after incubation with the *T. koningii* AUMC11521 for 7 days at 30 °C confirmed the two enzymatic hypothesized biodegradation pathway of OTA. According to the LC–MS/MS spectra, the detected *m/z* m/z 402.9, m/z 258.2, m/z 257.0, m/z 165.0, and m/z 150.2 were assigned to the separated OTA, OTα-amide, OTα, L-*β*-phenylalanine, and 3-phenylpropanoic acid, respectively. We suggest that OTA degradation occurred by two pathways (Fig. 6i, ii). First one occurred through the hydrolysis of the amide bond (Fig. 6i) via hydrolytic enzymes, such as CPA, carboxypeptidase PJ-1540, protease A, lipase A, ochratoxinases with amido-hydrolase activity, etc. which resulted in the production of L-*β*-phenylalanine molecule and a non-toxic OTα (Dobritzsch et al. 2014; Liuzzi et al. 2017). The second pathway involved the breakage between C-14 and the nitrogen atom of amine group as indicated by arrow in Fig. 6ii. This pathway resulted in the formation of OTα-amide and 3-phenylpropanoic acid. This is in accordance with Bittner et al. (2015). The cleavage of the amide bond (CO–NH) and NH–CH was confirmed as mentioned from FTIR spectra results. FTIR bands found at 1631 and 1600 cm^−1^ are assigned to C–N stretching vibrations. The four recorded products are less and non-toxic, so that this process is considered as detoxification. There is an additional pathway, occurred through the hydrolysis of the lactone ring by an ochratoxin-lactonase, resulted in the production of open ring OTA, which has the same toxicity as OTA, so that this process is not considered as detoxification but it is just break down of OTA molecules (Leitão and Enguita 2021). It is well known that the kidney is the first target for OTA. Bittner et al. (2015) studied the toxicity of OTα-amide using immortalized human kidney epithelial cells, a cell line known to be sensitive against OTA. The authors found that OTα-amide had no toxicity up to concentrations of 50 μM. Interestingly, 3-phenyl propionic acid is extensively used in cosmetics and pharmaceuticals as well as a preservative and flavoring agent in the food industry and it has a role as an antifungal agent (Korneev 2013).

## Conclusion

Overall, our endophytic strains of *T. koningii* (AUMC11519, AUMC11520 and AUMC11521) proved their efficient capability for degradation of OTA in vitro. The degradation products include OTα-amide, 3-phenylpropionic acid, OTα and phenylalanine which are much less toxic than OTA. The OTA degradation process by *T. koningii* strains is suggested to be enzymatic and CPA has a crucial role that could be used for OTA detoxification contamination in foods and food products. Furthermore, characterization of the genes that are responsible for the degradation of OTA would be a necessity.

## Supplementary Information

Below is the link to the electronic supplementary material.

Supplementary file1 (EPS 60 KB) Structure of OTA

## Data Availability

All relevant data are within the paper and its Supporting Information file.
